# Supplementary material for: Expert curation of the human and mouse olfactory receptor gene repertoires identifies conserved coding regions split across two exons
Source: BMC Genomics. 2020 Mar 3;21:196. doi: 10.1186/s12864-020-6583-3 (PMC7055050; doi:10.1186/s12864-020-6583-3)
Supplement: Supplementary file 1 — Additional file 1 Supplementary Fig. 1 | Same as Fig. 3 but for mouse protein-coding genes. Supplementary Fig. 2 | ORs have several isoforms per gene. Barplots of the number of genes with the indicated number of different transcript isoforms. Genes have been split into protein-coding (top) and pseudogenes (bottom), and by species (human on the left, mouse on the right). Supplementary Fig. 3 | Same as Fig. 4 but for the additional mouse OR genes that share a 5′ UTR exon with a neighbouring gene. mRNA, EST or PacBio clones supporting splice junctions between the two genes are indicated above the corresponding transcript. Supplementary Fig. 4 | Same as Fig. 4 but for the additional human OR genes that share a 5′ UTR exon with a neighbouring gene. mRNA, EST or PacBio clones supporting splice junctions between the two genes are indicated above the corresponding transcript. Supplementary Fig. 5 | Additional example of a split OR gene. On chromosome 7, Olfr682-ps1 was annotated as a pseudogene, but we identified an open reading frame (ORF) spanning two exons that codes for a 311 aa protein. This gene is a polymorphic pseudogene that, in the reference genome, contains a frameshift in the C-terminal domain (purple transcript); however, several mouse strains contain a 2 bp indel at position 105,126,541 that restores the correct frame. The splice junction and protein sequence are conserved in several mammals, including dog, cow and sheep. Olfr682-ps1 has a close paralogue, Olfr680-ps1, which shares 97% identity at the protein level. Whereas Olfr680-ps1 lacks transcriptional evidence, we used the conservation with Olfr682-ps1 and other mammals to annotate a full-length split transcript structure. [file 12864_2020_6583_MOESM1_ESM.pdf]

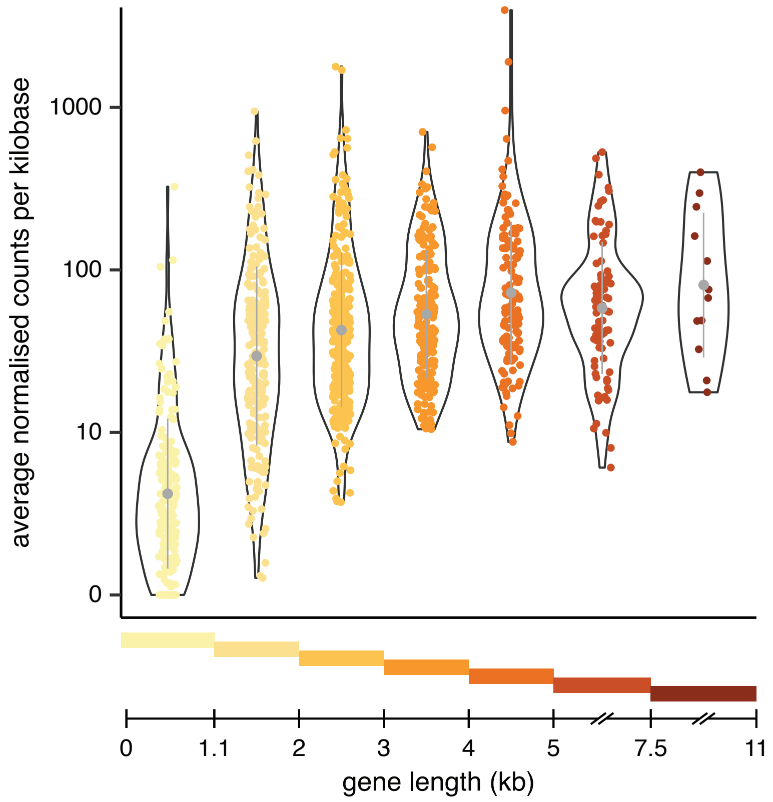

Supplementary Figure 1 l Same as Figure 3 but for mouse protein-coding genes.

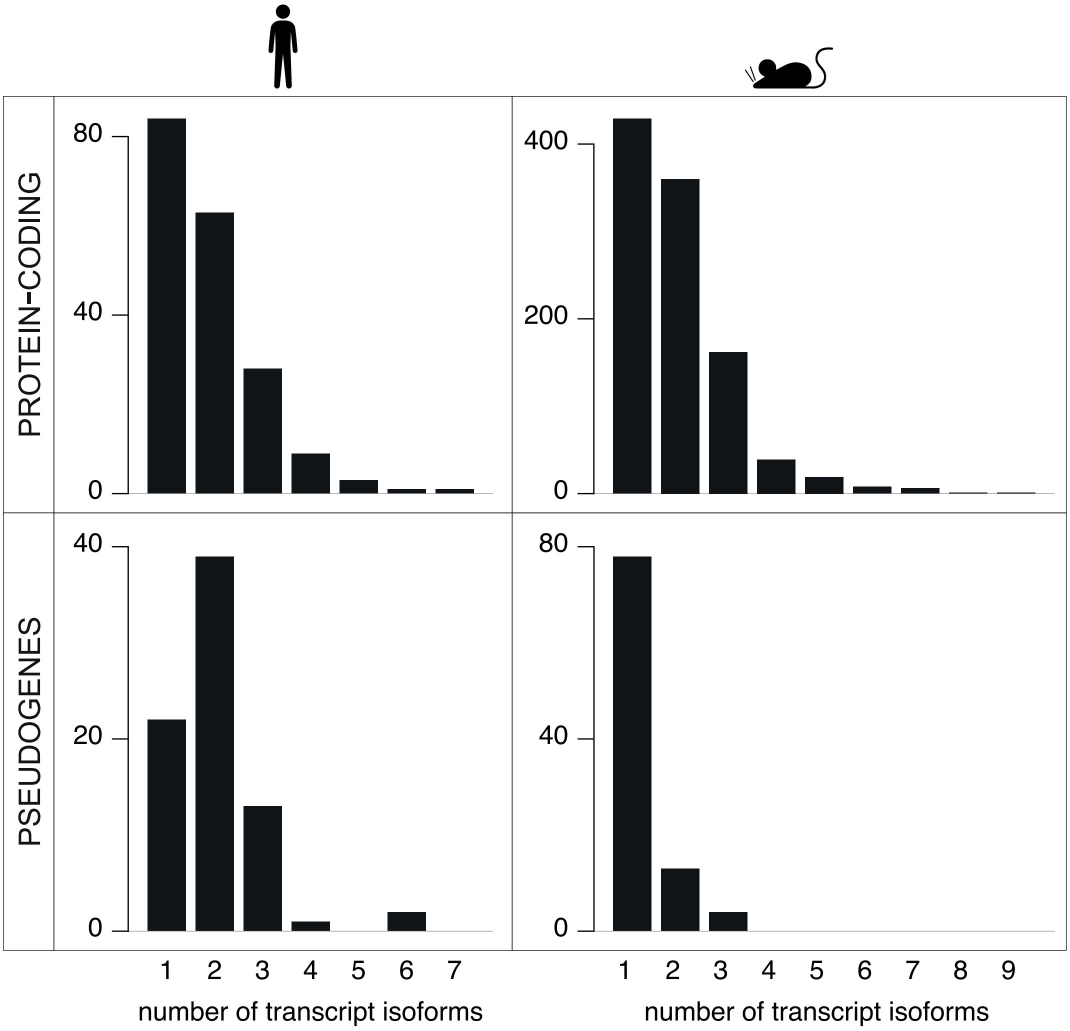

Supplementary Figure 2 I Olfactory receptors have several isoforms per gene. Barplots of the number of genes with the indicated number of different transcript isoforms. Genes have been split into protein-coding (top) and pseudogenes (bottom), and by species (human on the right, mouse on the left).

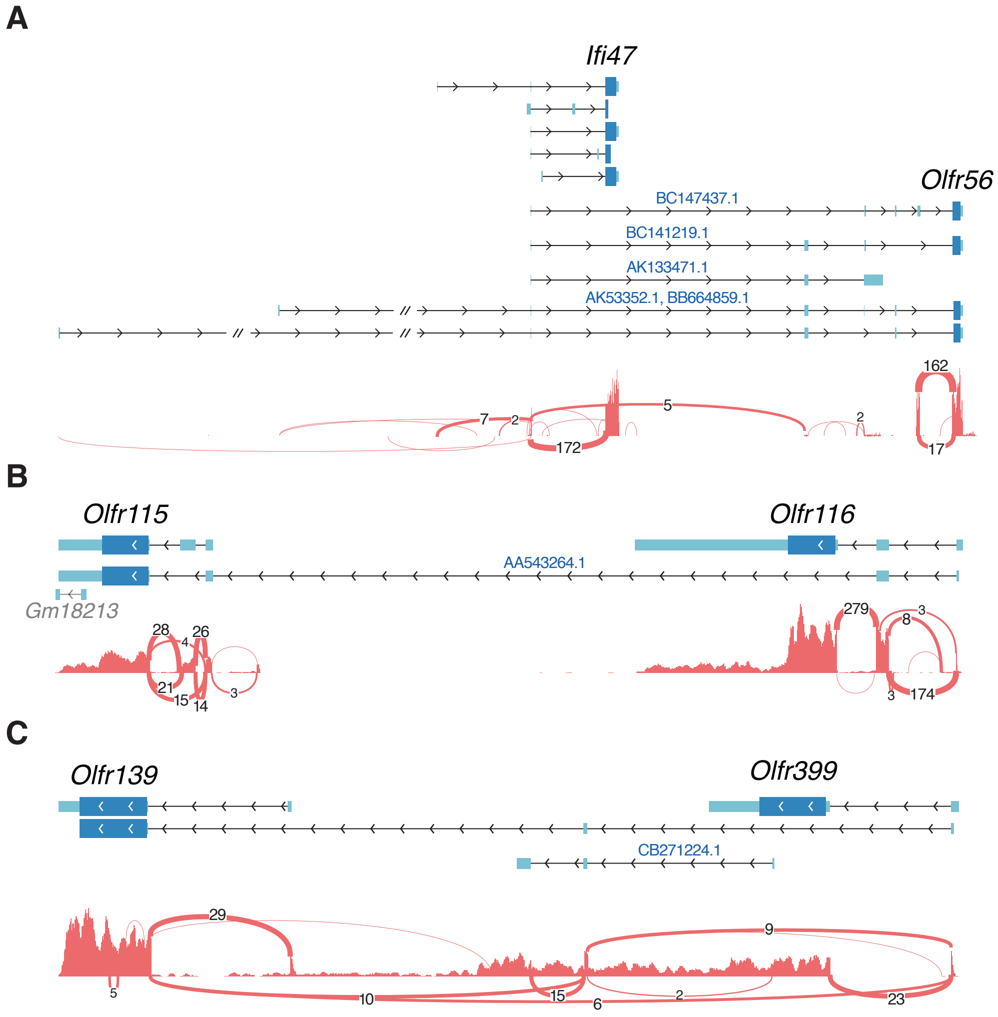

Supplementary Figure 3 I Same as Figure 4 but for the additional mouse ORs that share a 5' UTR with a neighbouring gene. mRNA, EST or PacBio clones supporting splice junctions between the two genes are indicated above the corresponding transcript.

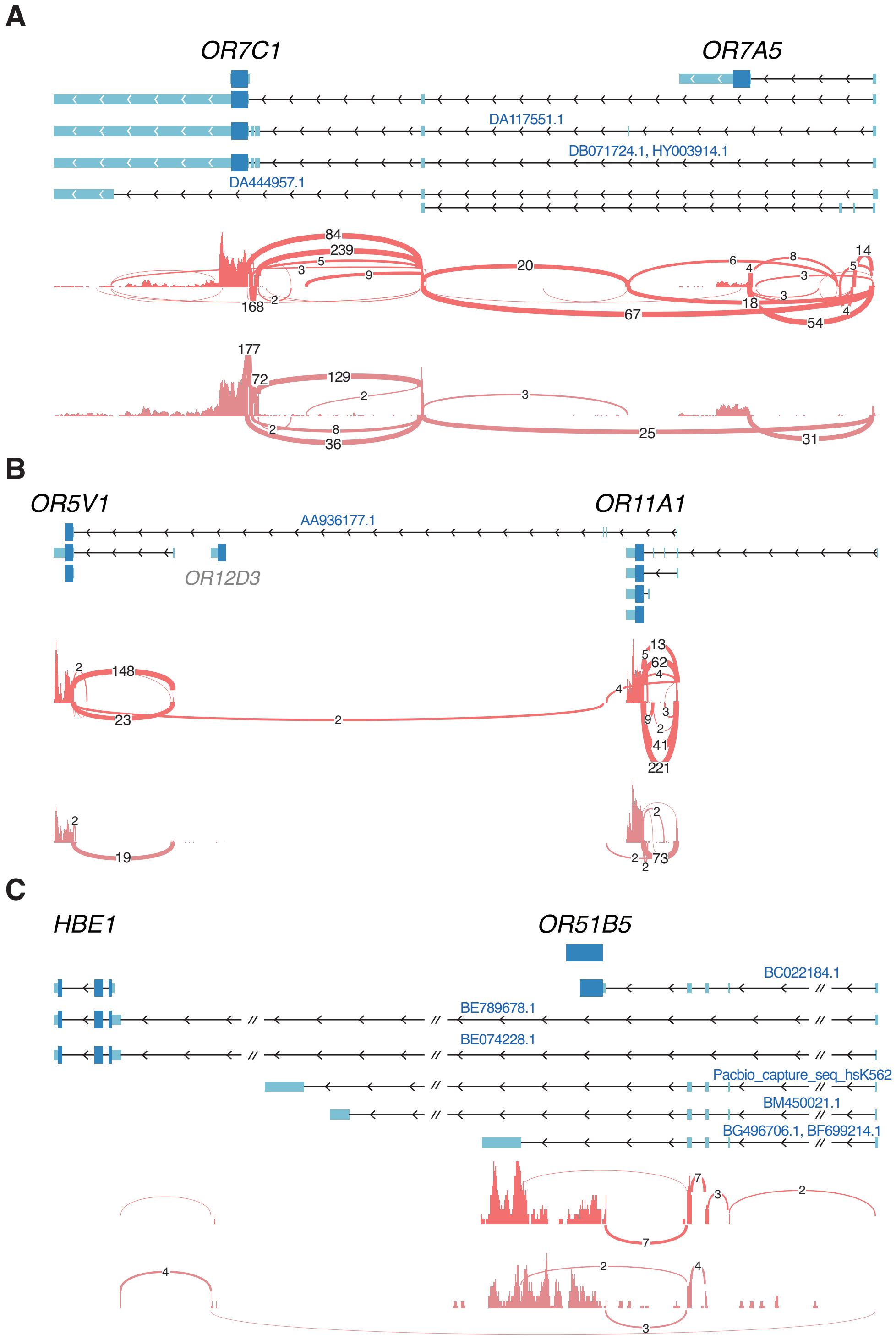

Supplementary Figure 4 I Same as Figure 4 but for the additional human ORs that share a 5' UTR with a neighbouring gene. mRNA, EST or PacBio clones supporting splice junctions between the two genes are indicated above the corresponding transcript.

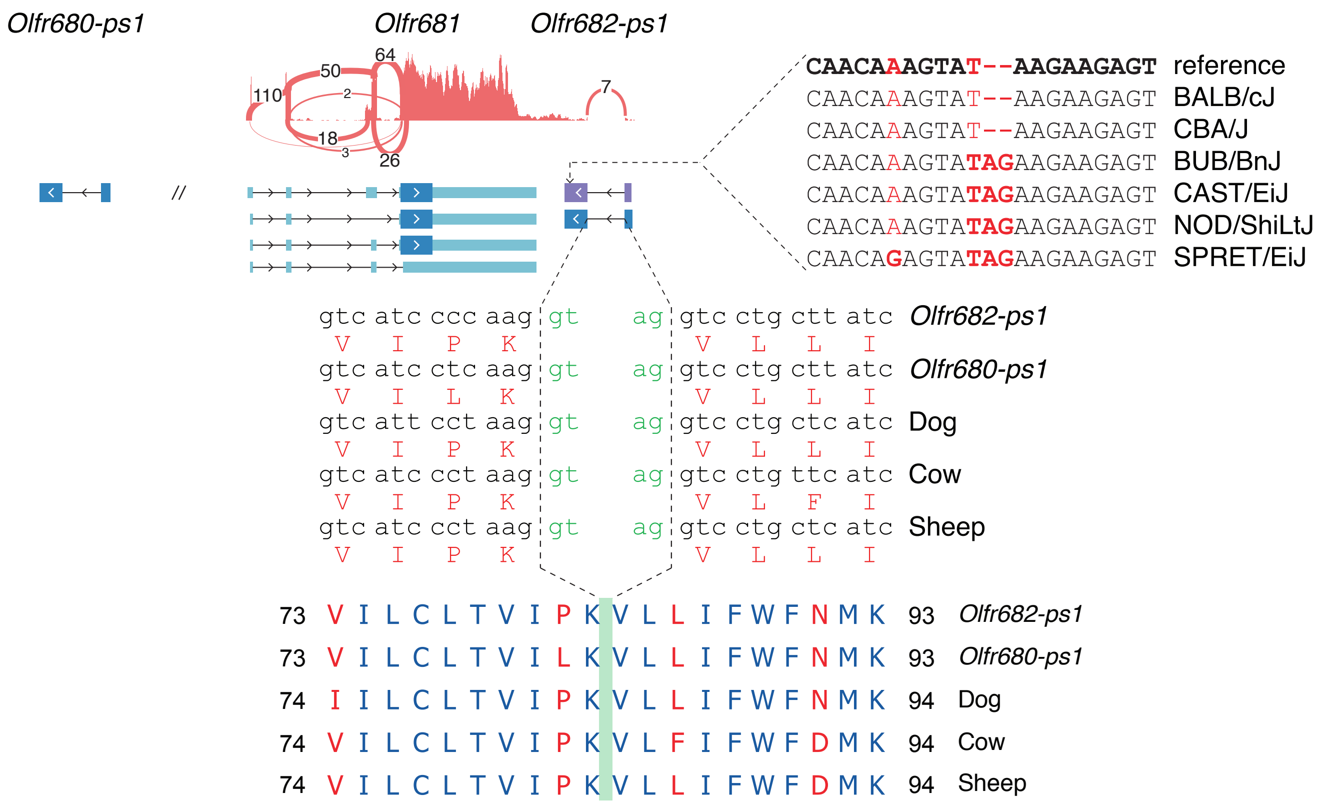

Supplementary Figure 5 I Additional example of a split OR gene. On chromosome 7, Olfr682-ps1 was annotated as a pseudogene, but we identified an open reading frame (ORF) spanning two exons that codes for a 311 aa protein. This gene is a polymorphic pseudogene that, in the reference genome, contains a frameshift in the C-terminal domain (purple transcript); however, several mouse strains contain a 2bp indel at position 105,126,541 that restores the correct frame. The splice junction and protein sequence are conserved in several mammals, including dog, cow and sheep. Olfr682-ps1 has a close paralogue, Olfr680-ps1, which shares 97% identity at the protein level. Whereas Olfr680-ps1 lacks transcriptional evidence, we used the conservation with Olfr682-ps1 and other mammals to annotate a full-length split transcript structure.
